# Advances in targeted retinal photocoagulation in the treatment of diabetic retinopathy

**DOI:** 10.3389/fendo.2023.1108394

**Published:** 2023-03-02

**Authors:** Zichun Lin, Aijun Deng, Ning Hou, Liyu Gao, Xushuang Zhi

**Affiliations:** Affiliated Hospital of Weifang Medical University, School of Clinical Medicine, Weifang Medical University, Weifang, China

**Keywords:** diabetic retinopathy, targeted retinal photocoagulation, retinal vascular disease, retina, laser, treatment

## Abstract

**Aim:**

Targeted retinal photocoagulation (TRP) is an emerging laser technology for retinal targeted therapy. TRP can specifically act on unperfused retinal capillaries and retinal intermediate ischemic areas, reduce damage to tissue perfusion areas and panretinal photocoagulation (PRP) complications or adverse events. In this regard, this review discusses the treatment options, efficacy, and latest progress of TRP for diabetic retinopathy (DR) based on randomized controlled trial (RCT), meta-analysis, case review, and other existing studies.

**Methods:**

In-depth research was conducted on articles about the proposal and development of TRP, its simple application in DR, and combined therapy. In order to review the new progress, application methods, effects, and prospects of TRP in the treatment of DR, the articles related to TRP in the databases of PubMed and Web Of Science since this century were comprehensively analyzed.

**Results:**

TRP is effective in treating DR and may become a substitute for PRP in the future. In addition, the treatment regimen of TRP combined with intravitreal injection of anti-vascular endothelial growth factor (anti-VEGF) drugs can also be used as a new therapeutic approach to expand the treatment regimen for the treatment of DR, and this combination therapy also has effects on other retinal vascular diseases.

**Conclusions:**

With the advancement of technology, TRP has been continuously applied in clinical practice, and its potential benefits have opened up broad prospects for the treatment of DR. The combination therapy of TRP and anti-VEGF is expected to become a new option for patients with DR an retinal diseases.

## 1 Introduction

**Figure 1:**
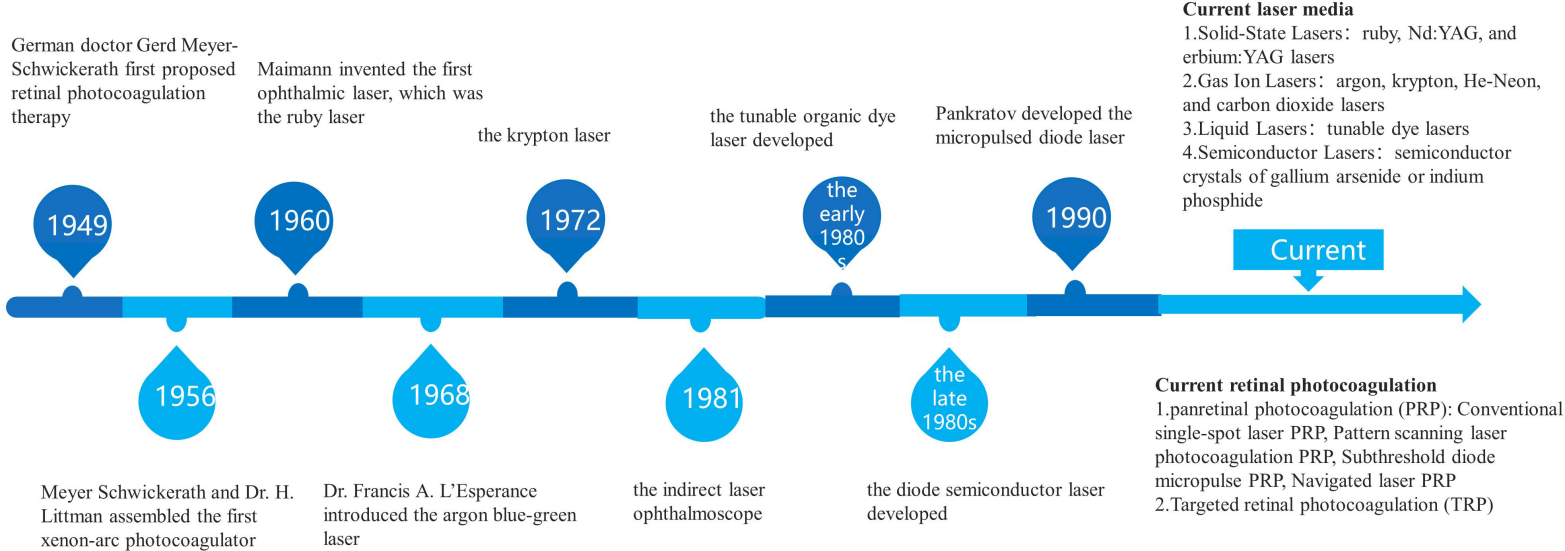
Advances in targeted retinal photocoagulation in the treatment of diabetic retinopathy.

The effects of laser interaction with retinal tissue have long been mentioned, in the 1940s when German ophthalmologist Meyer-Schwickerath pioneered retinal photocoagulation (1, 2). For more than 40 years, retinal photocoagulation has been used as the standard choice for the treatment of retinal vascular diseases and complications, mainly including proliferative diabetic retinopathy (PDR), diabetic macular edema (DME), retinal vein occlusion (RVO) and retinal tears, etc (**Figure 1**).

The scope of traditional PRP is mainly distributed in the middle and peripheral part of the retina, 1.5-2 optic disc diameters (DD) posteriorly from the optic disc and 2 DD temporally from the fovea, bounded by the superior and inferior vascular arches; forward to the ampulla of the vortex vein (or equator). Currently, PRP is the gold standard for the treatment of extensive areas of non-perfusion (suggesting severe retinal ischemia) (Diabetic Retinopathy Research Group 1981) (3), as well as the main method for the treatment of severe nonproliferative diabetic retinopathy (NPDR) and PDR. However, due to the photochemical damage of the laser, panretinal laser photocoagulation causes more damage to the ocular tissue, and its side effects include hemorrhage, choroidal detachment, acute angle-closure glaucoma, decreased color vision and contrast sensitivity, night blindness, lens burn (4–6) and permanent retinal scarring resulting in blind spots, etc. The occurrence of these complications is closely related to laser parameters such as increased duration and power and intensive treatment in a single session, which all lead to increased diffusion of thermal energy within the retina and choroid (7).

## 2 Materials and methods

**Figure 2:**
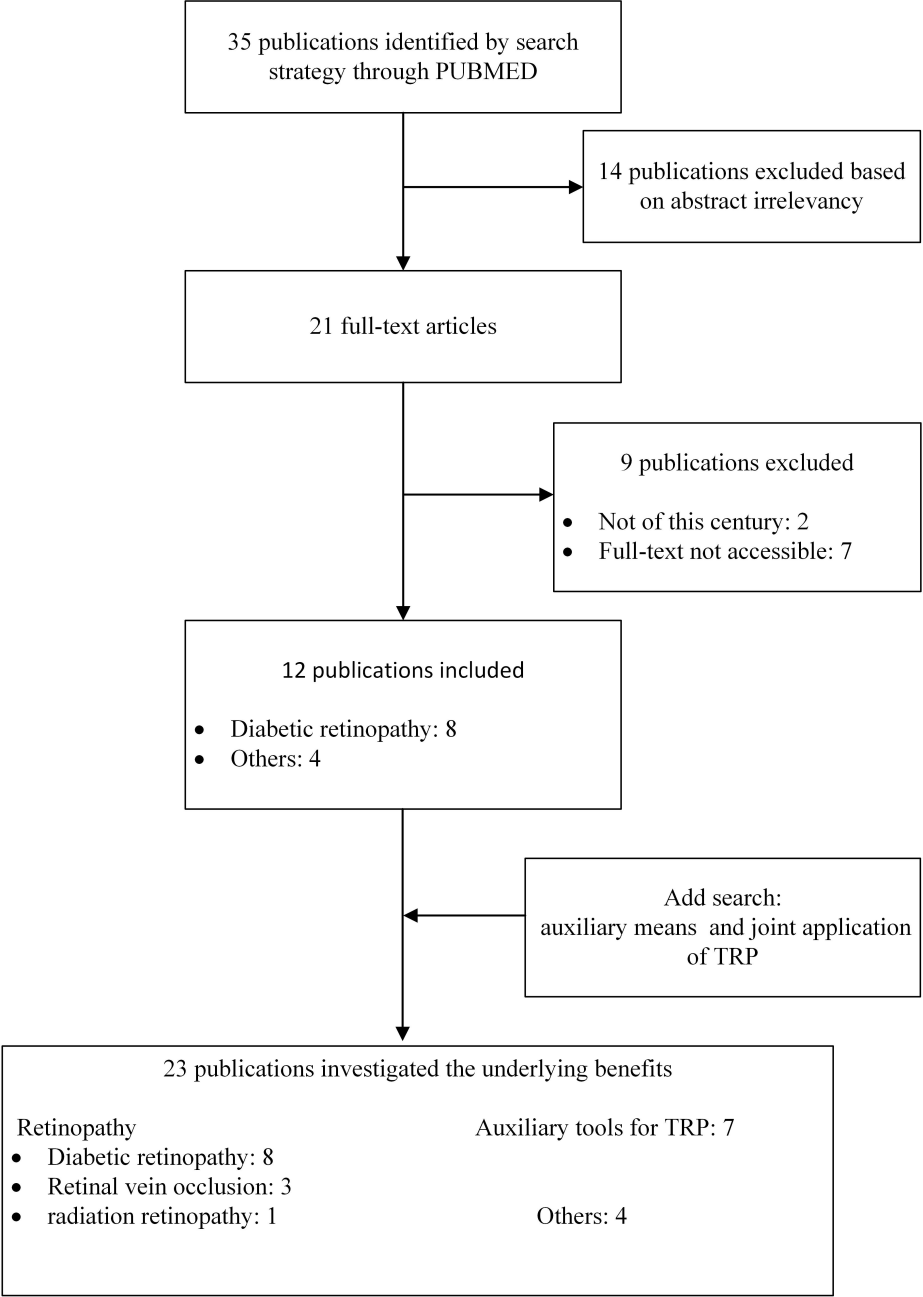
Flow diagram of systematic search.

The databases of PubMed, Web of Science were searched using the following keyword combinations: Targeted Retinal Photocoagulation, TRP, Diabetic Retinopathy, Retinopathy. Related articles discuss the effects of various treatment options of TRP in DR and other common retinopathy through RCT, meta-analysis, case review and other research methods. Inclusion criteria included full text, English and publications from this century. Articles were initially selected by searching for titles and abstracts. The full text of the papers was reviewed and papers with titles or abstracts that did not fit the purpose of this review or did not provide sufficient data for a full evaluation were excluded. To complement the search, citations to relevant articles were also collected, and we got 23 qualified publications (**Figure 2**). We grouped all obtained studies into application of TRP in DR and other retinopathy (RVO and radiation retinopathy), summarized and analyzed the main research contents of TRP in treating DR in chronological order (**Table 1**). To try to explore the effects and potential benefits of these treatment schemes.

**Table 1:** The historical process of the treatment of PDR with TRP.

Time	Magazine	Type	Ideas	Content	Conclusion
2009 (8)	Semin Ophthalmol.	Case Reports	PRP may excessively damage the retina, so consider using UWFFA and TRP to reduce complications	Two cases of using UWFFA to guide TRP to the NPAs of retinal capillary were reported for the first time	When TRP is used in combination with UWFFA, it can cause the regression of neovascularization in diabetes and minimize the amount of laser treatment applied to the retina
2012 (9)	Clin Ophthalmol.	Clinical Trial	Accurate positioning during laser treatment may help to improve the clinical results of laser treatment	TRP was performed on 54 patients with DME by using navigation laser photocoagulation system Navilas	The laser navigation effect is consistent with the target area in positioning, and the pain associated with treatment is significantly lower than PRP
2013 (10)	Br J Ophthalmol.	Randomized Controlled Trial	In order to reduce the adverse effects of laser treatment, many experts began to use the laser with lighter burn and lower intensity than the original provisions of ETDRS to treat patients	The short-term effects of PRP and TRP on macular thickness were compared	Local laser treatment did not increase the macular thickness or any serious adverse events in the eye in a short time
2013 (3)	Acta Ophthalmol.	Clinical Trial	Compared with PRP, TRP may prevent well-perfused tissue from laser-induced tissue scar formation	TRP of 28 patients with PDR at the initial stage of treatment with WFFA	TRP, a laser technology that only targets the local ischemic retina, can reduce the potential long-term damage associated with the expansion of long-pulse PRP burn
2014 (11)	Invest Ophthalmol Vis Sci.	Randomized Controlled Trial	Find a technology to reduce DME recurrence after intravitreal injection of anti-VEGF drugs to avoid repeated injection	Apply IVB or IVB+TRP to 52 DME patients	TRP for NPAs can effectively maintain the reduced CRT after IVB
2018 (12)	Int Ophthalmol.	Randomized Controlled Trial	ETRP procedure to treat areas of capillary non-perfusion and intermediate ischemic zones posterior to the equator as well as the entire retina anterior to the equator	Apply PRP or ETRP to 234 PDR patients	ETRP is a sensible substitution for CPRP to induce PDR regression.
2018 (13)	Ophthalmology.	Clinical Trial	To test the hypothesis that WFFA eguided TRP significantly reduces the number of required anti-VEGF injections	Apply IVR or IVR+TRP to 29 DME patients	There was no significant evidence that the combination of leizumab and TRP was more effective compared to Leizumab alone.
2021 (14)	Doc Ophthalmol.	Randomized Controlled Trial	Compared with PRP, the leakage area of neovascularization in PRP+IVR is reduced, and more focal TRP is considered to reduce the loss of retinal function	Applying IVR+PRP or IVR+TRP to 28 PDR patients	The reduction level of retinal neovascularization leakage area by two laser modes is similar in one year

PDR, proliferative diabetic retinopathy; TRP, targeted retinal photocoagulation; PRP, panretinal photocoagulation; UWFFA, ultra-wide-angle fluorescein fundus angiography; DME, diabetic macular edema; ETDRS, early treatment of diabetic retiopathy study; WFFA, wide-angle fluorescein fundus angiography; anti-VEGF, anti-vascular endothelial growth factor; IVB, intravitreal bevacizumab; NPAs, non-perfusion areas; CRT, central retinal thickness; ETRP, Extended TRP; CPRP, central panretinal photocoagulation; IVR, intravitreal ranibizumab.

## 3 Results

### 3.1 Progress of TRP

In 2009, Reddy et al. improved on the basis of panretinal laser photocoagulation and designed a peripheral laser strategy assisted by ultra-wide-angle fluorescein angiography (8),to reduce the damage to the retina caused by panretinal laser photocoagulation, which is also the prototype concept of targeted retinal laser photocoagulation.

TRP is a laser technology aimed at the peripheral non-perfusion and ischemic areas of the retina, and the laser area is determined according to the degree and progression of the patient’s retinopathy. To ensure coverage of the entire ischemic area and its margins (usually suspected of having high-density or leaking microaneurysms), the laser extends 1-2DD of the laser scan over the peripheral ischemic and non-perfused areas. Compared with panretinal laser photocoagulation, targeted retinal laser photocoagulation is more targeted to the treatment area and has fewer laser points, so it can minimize the amount of laser treatment and reduce the occurrence of ocular adverse events caused by retinal photocoagulation rate.

The value of ultra-wide-angle fluorescein fundus angiography (UWFFA) and ultra-wide-angle swept-frequency optical coherence tomography (WF SS-OCTA) in TRP. Since the key of TRP treatment is to accurately treat the non-perfused and ischemic areas of the retina, accurate positioning of the entire ischemic area of the retina is the primary condition for the successful treatment of targeted lasers. Visualization of the entire retina is fundamental to assess non-perfused areas, vascular leakage, microvascular abnormalities, and neovascularization, and ultra-wide-field (UWF) imaging improves retinal visualization by up to 82% in a single image (15).

Fluorescein angiography (FA) has been used for 50 years in the evaluation of superficial retinal vascular abnormalities in different stages of DR. In order to expand the inspection field, people currently mainly choose to use wide-field (>30° and >200°) and UWF (≥200°) fundus angiography and UWFFA (16). Since UWFFA can capture a 200° area and can view the posterior pole, central peripheral and peripheral fundus, in another study it was confirmed that the capillary non-perfusion (CNP) area detected by UWFFA in patients with DR was 3.9 times that of conventional angiography (17). Thus, UWFFA is an important aid for the successful implementation of targeted lasers.

With the advent of optical coherence tomography angiography (OCTA), it has become possible to obtain highly detailed depth-resolved images of retinal and choroidal microvascular abnormalities. While early OCTA focused on viewing the macular region, WF SS-OCTA significantly increases the field of view (FOV) at the retinal surface, and WF SS-OCTA has been shown to be effective in identifying diabetic retinopathy (18, 19). Two case analyses reported the correspondence and superiority of WF SS-OCTA relative to UWF FA in longitudinal assessment of retinal nonperfusion (RNP) in PDR, with higher detection rates in NP areas after anti-VEGF drug injection treatment (20), and provided important insights into the stability of retinal ischemia after PRP (21). However, there is no clinical report on TRP assisted by WF SS-OCTA.

### 3.2 Clinical application of TRP

#### 3.2.1 TRP for DR

##### 3.2.1.1 TRP therapy

DR is the main cause of blindness in diabetic patients, and the main pathological change in blindness is neovascularization. At present, the main method for the treatment of retinal neovascularization is retinal photocoagulation. Since this century, the research of TRP has increased year by year. Researchers have comprehensively explored the therapeutic effect and safety of TRP on DR through combined treatment, comparative control and other methods (**Table 2**). In 2009, the University of California reported two cases of UWFFA-assisted targeted laser treatment of diabetic retinopathy (8). For both patients, 532 nm laser was accurately applied to the ischemic area displayed and clearly demarcated by UWFFA for a total of 1000-1400 times. This treatment technology first entered the public eye as an individualized treatment plan: retinal angiography found that TRP successfully regressed retinal neovascularization in two patients. At 9-month follow-up, the patients’ vision was maintained and there was no recurrence of neovascularization and macular edema. In a clinical trial reported in the United Kingdom in 2013 (3), 20 newly treated patients with PDR were treated with TRP alone. The results of the study show that in the short term (12 weeks to 24 weeks), its clinical efficacy and safety are worthy of recognition:76% of patients experienced PDR regression at week 12; patients’ vision and visual field did not worsen or even improved; macular thickness was significantly reduced (mean baseline 251 μm, 12.1 μm at 24 weeks); disease progression was slow, and no TRP-related ocular adverse events occurred during the study period.

**Table 2:** Summary of studies targeted retinal photocoagulation for diabetic retinopathy.

Study	Patients	Targeted laser range	Outcomes of interest (primary and secondary)	Follow-up period	Main conclusions
Shantan Reddy et al. (8)	A middle-aged man with NPDR in both eyes and a middle-aged woman with PDR in the left eye	retinal ischemic area	Ultra wide field fluorescein angiography	9 months	TRP successful led to the regression of the retinal neovascularization
Mahiul M K Muqit (3)	Treatment-naive PDR (n=20)	areas of peripheral retinal capillary non-perfusion and intermediate zones of perfused and non-perfused retina	PDR grade; CRT; MD; standard V; ETDRS VA	24 weeks	TRP has a satisfactory short-term safety profile and is a promising treatment option with no deterioration in CRT, VA, or VF in the affected eye after short-term treatment
Mahiul M K Muqit (10)	Treatment-naive PDR (n=24)	area of capillary non-perfusion from the ora serrata up to 1 DD into perfused retina	Primary Outcome Measures: change in CRT on OCT Secondary Outcome Measures: OCT peripapillary NFL thickness; PDR disease regression on optos angiography; VF; VA	12 weeks	high-density 20-ms TRP using 2500 burns did not produce increased macular thickness or any ocular adverse events during the short-term
Marcus Kernt (9)	DME (n=54)	navigated, semi-automatic pattern laser application	visual analog scale directly; Navilas color images	1 months	treatment-related pain following Navilas laser photocoagulation was significantly lower than pain following conventional laser treatment
Homayoun Nikkhah (12).	naïve early or high-risk PDR (n=234)	areas of capillary non-perfusion and intermediate ischemic zones posterior to the equator as well as the entire retina anterior to the equator.	Primary Outcome Measures: early PDR regression, specified as reduction in retinal neovascularization based on WFFA at 3 months. Secondary Outcome Measures: BCVA; CMT changes.	3 months	TRP may be an appropriate alternative to PRP in PDR regression at least through 3 months
David M Brown (13)	DME (n=29)	areas of nonperfused peripheral retina plus a 1edisc area margin	Primary Outcome Measures: mean change in ETDRS BCVA from baseline and number of intravitreal injections administered Secondary Outcome Measures: percentage of patients gaining or losing 15 ETDRS letters or more from baseline, mean change in CRT, mean change in peripheral visualfield as measured by GVF testing, and incidence and severity of adverse events.	36 months	Combination therapy did not reduce treatment burden and improve vision compared with drug injection alone, but may reduce the development and incidence of neovascular complications
Yoshihiro Takamura (11)	DME (n=52)	nonperfused areas (NPAs)	BCVA;CRT	6 months	TRP for NPAs was effective to maintain the reduced CRT after grid/focal photocoagulation and IVB for patients with DME
Luiza Toscano (14)	adult patients with treatment-naive PDR and a BCVA better than 20/800 (n=23)	retinal ischemic areas	Primary Outcome Measures: FLA of active new vessels Secondary Outcome Measures : BCVA;CSFT; Number of Ranibizumab Intravitreal Injections; Electroretinography	48 weeks	PIR+IVR or PRP+IVR are comparable strategies regarding FLA control in PDR and led to similar retinal function impairment

PDR, proliferative diabetic retinopathy; CRT, central retinal thickness; MD, mean deviation; TRP, targeted retinal photocoagulation; VF, visual fields; VA, visual acuity; NFL, nerve fibre layer; PRP, panretinal photocoagulation; UWFFA, ultra-wide-angle fluorescein fundus angiography; DME, diabetic macular edema; ETDRS, early treatment of diabetic retiopathy study; WFFA, wide-angle fluorescein fundus angiography; anti-VEGF, anti-vascular endothelial growth factor; IVB, intravitreal bevacizumab; NPAs, non-perfusion areas; IVR, intravitreal ranibizumab; FLA, fluorescein leakage area; BCVA,best corrected visual acuity.

##### 3.2.1.2 Ultra-wide-angle assisted guidance TRP

The visual field of fluorescein angiography of the standard fundus camera is 30 – 60°. It is necessary to take different images at different times in the study to image the periphery of the retina. This makes it very difficult to accurately delineate retinal capillary non-perfusion and accurately apply photocoagulation within the ischemic boundary. UWFFA is a very useful tool in the diagnosis, staging, management and treatment of DR (22). UWFFA can display more retinal pathology even in eyes judged as normal by 7 standard field (7SF) (16). In 2008, Friberg et al (23). first reported the feasibility of UWFA in 30 eyes of 30 DR patients. Compared with the standard system, they observed that UWFA allowed imaging of a larger area of retinal surface (8.7 ± 1.6 vs 3.4 ± 0.76DD, P<0.001) and retinal ischemia (16.9 ± 15 vs 3.4 ± 4.26 sectors, P<0.05), although the image quality was reduced. Then in 2009, the first case report (8) of TRP and UEFFA mentioned that under UWFFA, the non-perfusion area and its boundary of the patient’s retinal capillaries could be identified very accurately, providing a clearer and more discernible field of vision for the application of TRP. UWFFA can initially and accurately locate the non-perfusion area of retinal capillaries in a 200° field of vision at the early stage of application. In 2013, Muqi et al. (3) evaluated the effect of UWFFA-guided TRP in the eyes of 28 PDR patients. At 12 weeks, 76% of patients had PDR regression, and 37% had complete disease regression at 24 weeks. They found that the thickness of the central retina decreased significantly over time, measured by optical coherence tomography (22). With the gradual maturity of the UWFFA program, as an assistant retinal examination tool, it has been more and more widely used in clinical work. However, the specific use standards for its joint application with TRP still need to be further clarified through large-scale research in the future.

##### 3.2.1.3 Compared with panretinal photocoagulation

The primary difference between targeted retinal laser and panretinal laser photocoagulation is that the narrowing of the laser range results in a significant reduction in the number of laser spots on the retina. In all randomized controlled clinical trials, regardless of whether the targeted laser range is specified as “optos-guided TRP treatment covered the area of capillary non-perfusion from the ora serrata up to 1 DD into perfused retina (10)”, “navigated, semi-automatic pattern laser application was conducted based on the treatment plan (9)” or “capillary non -perfusion and intermediate ischemic zones posterior to the equator as well as the entire retina anterior to the equator (12)”, the TRP has significantly fewer laser spots (9, 12) or lower power (10). In this regard, the visual analog scale was used to quantify the degree of pain in the treatment of patients, and it was found that the pain of TRP was significantly lower than that of PRP.

For the improvement of visual function, both treatment modalities improved visual acuity and mean visual field defect in the short term (4-12 weeks) but there was no significant difference between the two (10); in the medium and long term (at least 3 months)) The mean best-corrected visual acuity (BCVA) after treatment was significantly decreased, and the difference was not significant (12). DME is a serious complication of DR and one of the main causes of visual loss in DR patients, so central macular thickness (CMT)/central retinal thickness (CRT) is also an important research parameter. CMT decreased in the short term (4-12 weeks) after PTR treatment, and was not significantly different from PRP; although a clinical trial (NCT01232179) (12) reported that TRP or PRP alone was used to treat patients with early or high-risk PDR after 3 months There is an increase in CMT, and CMT thickening after laser treatment has been demonstrated in many other studies (9, 24–26), but there is no difference in results between the two laser modalities. In terms of safety, there were no serious ocular complications or adverse events immediately after TRP treatment, short-term or even mid-to-long term, and there were no signs of intraretinal hemorrhage, vascular damage or traction retinal detachment in the laser treatment area. There is also no adjustment to the treatment regimen for patients treated with TRP, such as switching to PRP.

Comparing the efficacy and safety of the two laser methods, after analyzing many research parameters such as the number of laser points, visual function, UWF, pain level, prognosis, and adverse events, there may be no difference in the efficacy of the two on DR. But TRP reduces retinal damage and preserves more healthy retinas due to fewer laser points, which also improves patient comfort. Therefore, TRP may be a future alternative to PRP to some extent (27)

##### 3.2.1.4 Combination therapy of TRP and vitreous anti-VEGF drug injection

With the research on the pathophysiology of retinal vascular diseases, the use of anti-VEGF drugs for the treatment of retinal vascular diseases has become more and more popular. Such drugs can bind tightly to VEGF to reduce vascular permeability and then inhibit the formation of new blood vessels. Anti-VEGF is currently the gold standard treatment for veno-occlusive macular edema, especially BRVO (28). However, the short half-life of the drug causes its concentration in the eye to drop rapidly and the concentration in the vitreous cavity is unstable and the treatment effect is short. Retinal neovascularization and macular swelling are prone to recur after a single injection of anti-VEGF drugs (13). Therefore, frequent intravitreal injections of anti-VEGF drugs are required to control macular edema, which increases the likelihood of adverse events such as endophthalmitis and retinal detachment. Researchers have found in clinical trials that Through the combined application of vitreous drug injection and targeted laser therapy, the use of targeted laser can effectively reduce the frequency of drug injections or improve the prognosis, which may achieve the purpose of reducing the number of patient visits, costs and the risk of adverse events.

Current studies have suggested that anti-VEGF drugs can reduce retinal thickness, improve DME, and improve vision in the treatment of DR, but require repeated injections for optimal results. In this regard, in order to reduce the number of injections of anti-VEGF drugs and reduce the occurrence of adverse events, researchers considered the combined application of TRP and intravitreal injection of anti-VEGF drugs. At present, two controlled clinical trials have been completed in Japan (UMIN000007566) and the United States (NCT01552408), involving a total of 81 DME patients. Visual function and CRT were mainly compared between patients receiving TRP and anti-VEGF drugs in combination with patients receiving anti-VEGF drugs alone. However, the results of the two trials are contradictory: after a 6-month study by the Japanese team, it was found that the average best corrected visual acuity (BCVA) of the combined treatment group improved more significantly, And the CRT of the patients in the injection-only group increased to a higher degree at the 3rd, 4th, and 5th months (11);however, after a 3-year Randomized DAVE Trial, the US team found that the combined treatment group was not better than the single injection group in terms of BCVA, visual field, CRT, and the number of injections, but in In terms of safety, there were 5 new cases of new PDR-related neovascularization in the drug injection group but no endophthalmitis in the combined treatment group (13). The difference in results between the two groups may be related to the inclusion criteria, dosing schedule, and sample size. Compared with the US study, the participants in the Japanese trial were injected with bevacizumab (1.25 mg IV bevacizumab: 0.3 mg IV ranibizumab), which may explain the difference in results between the two groups.

In addition, a controlled clinical trial (NCT03904056) (14) reported by the São Paulo State University research team in 2021 compared the effect of PRP combined with intravitreal injection of ranibizumab (IVR) and TRP combined with IVR in the treatment of PDR. The results showed that there were no significant changes in BCVA and central subfield thickness (CSFT) between the two groups; the fluorescein leakage area of active new vessels (FLA) was significantly reduced in the two groups, but there was no between-group difference. In addition, we checked on the International Clinical Trials Registry website (ClinicalTrials.gov) that the Cairo University research team has initiated a RCT (NCT04674254),to compare the changes of macular area in PDR patients receiving anti-VEGF therapy combined with TRP or PRP therapy, and we will continue to pay attention to this.

Overall, TRP helps to improve DME recurrence after vitreous drug injection, additional laser may help to suppress VEGF production in the ischemic area, and combination therapy of TRP and anti-VEGF drugs may help reduce the frequency of vitreous drug injections, patient treatment costs, number of clinic visits, and adverse events (eg, endophthalmitis, retinal detachment).

#### 3.2.2 TRP of other retinopathy

In addition to DR, the researchers also studied the therapeutic effects of TRP on other retinopathy. We mainly analyzed the effects of TRP on RVO and radiation retinopathy (**Table 3**). Studies have found that RVO is the second most common retinal vascular disease, and macular edema is a common complication of this disease and the main cause of reduced vision. Therefore, improving macular edema is a key link in the treatment of this disease. The researchers selected patients with RVO and macular edema (ME) who met the treatment criteria to conduct individualized treatment for a single case or controlled clinical trials with different durations of 6 months, 9 months, and 12 months have been carried out successively (28–30, 32).A total of 101 patients were enrolled, and the efficacy and safety of TRP combined with anti-VEGF drug injection and drug injection alone were compared.

**Table 3:** Summary of studies targeted retinal photocoagulation for retinal vein occlusion and radiation retinopathy.

Study	Patients	Targeted laser range	Outcomes of interest (primary and secondary)	Follow-up period	Main conclusions
Siddhi Goel (28)	treatment naïve BRVO with ME (n=32)	the CNP areas and at the junction of ischemic and nonischemic areas, extending anteriorly up to ora serrata	VA; CSFT; the number of injections required with a minimum follow-up of 9 months	9 months	TRP reduced the number of ranibizumab injections in patients with BRVO macular edema, while maintaining similar benefits to the combination arm in improving BCVA, CSFT, and contrast sensitivity
Soonil Kwon (29)	RVO with recurrent ME (n=30)	areas of retinal non-perfusio outside of the macula, no closer than two disc diameters from the optic nerve head	Primary Outcome Measures: Total Number of Intravitreal Injections Over a 12 Month Period;VA Secondary Outcome Measures: Retinal Ischemia; Foveal Avascular Zone; Adverse Events; Neovascularization of the Iris, Optic Nerve and Elsewhere; Central Foveal Outcome; Aqueous VEGF Levels;VF	12 months	The role of TRP, particularly to NPAs and PMA, warrants further investigation in recalcitrant RVO-associated ME
Yoko Tomomatsu (30)	BRVO with ME (n=38)	NPAs 3000μm away from the centre of the fovea	BCVA;CRT	6 months	RP of NPAs reduced the amount of ME recurrence following IVB compared to IVB alone
Hannah J. Yu (31)	radiation-related ME (n=40)	areas of peripheral retinal ischemia as defined by wide-field angiography	Primary Outcome Measures: BCVA Secondary Outcome Measures: The Mean Number of Intravitreal Injections; Percentage of Subjects With Retinal Hemorrhage; Percentage of Subjects With Intraretinal Exudates on Fundus Examination; Mean Change in Central Mean Thickness Compared to Baseline	104 weeks	Combination therapy did not result in significant visual and anatomical improvement over monthly medical therapy alone, but compared with history, serious complications were prevented

BRVO, retinal vein occlusion; ME, macular edema; TRP, targeted retinal photocoagulation; VA, visual acuity; CSFT, central subfield thickness; VEGF, vascular endothelial growth factor; VF, visual fields; RVO, retinal vein occlusion; BCVA, best corrected visual acuity; CRT, central retinal thickness; RP, retinal photocoagulation; IVB, intravitreal bevacizumab; NPAs, non-perfusion areas.

First, all patients showed significant improvement in visual function, in terms of BCVA: the mean increase in Early Treatment of Diabetic Retiopathy Study (ETDRS) letters in the RBZ group in the comparative trial with ranibizumab (RBZ) was 25.7 ± 8.2 (95% CI: 21.5–29.9), RBZ + TRP group was 23.38 ± 7.6 (95% CI: 19.3–27.4) (28); in the comparative trial of intravenous bevacizumab (IVB), the BCVA of the IVB+TRP group gradually improved and reached a significant level at 6 months, However, there was no significant improvement in the IVB group (30). In addition, the mean contrast sensitivity and visual field of the patients were significantly improved under both treatment regimens (28), and there were no disease progression or adverse events related to TRP or intravitreal injection, such as infectious ophthalmia, intravitreal hemorrhage, neovascularization, retinal detachment, etc. Second, patients’ mean CRT (or CMT) continued significant improvement (28–30). Finally, the combined application of TRP can reduce the number of injections as needed: the number of additional injections of bevacizumab in the IVB group was significantly greater than that in the IVB+TRP group (1.58 ± 0.69:0.83 ± 0.62); the RBZ group was also more frequent than the RBZ group. + TRP group (5.76 ± 1.3:4.06 ± 0.99), the above differences were all statistically significant. Therefore, compared with single drug injection, the combination therapy with TRP can achieve similar efficacy or even better prognosis on the basis of Reduce the number of injections, and reduce the development and recurrence of ME caused by RVO.

We also found on the International Clinical Trials Registry website (ClinicalTrials.gov) that the research team at the University of Leipzig has initiated a clinical trial comparing injection of ranibizumab with or without targeted photocoagulation in the treatment of central RVOwith macular edema (NCT04444492), which is expected to be completed in 2024, and the author will continue to pay attention to the progress and results of the trial.

Radiation retinopathy is a common, progressive visual side effect of ophthalmic radiation therapy for intraocular or orbital tumors. It typically presents as late-onset disease of the retinal vasculature between 6 months and 3 years after radiation therapy, with clinical signs of macular edema, cotton wool spots, neovascularization, and/or vitreous hemorrhage, which has a serious impact on the vision of patients (31, 33–36). Therefore, Hannah J Yu et al. (31) also tried to treat the disease with TRP. This randomized controlled clinical trial (NCT0222610) included 40 patients with radioactive ME, and compared the efficacy and tolerance of intraocular injection of RBZ and RBZ+TRP in the treatment of patients with radioactive retinopathy. After 2 years of treatment and follow-up, it was found that the average ETDRS BCVA of patients improved after 1 year, but the effect of combined treatment was not significantly better than that of injection alone. The trial found that additional TRP did not bring significant visual and anatomical improvement to patients, but it may prevent serious complications during treatment. Because there are few clinical trials related to this disease at present, a large number of clinical trials are still needed to confirm the efficacy and safety of TRP for this disease.

## 4 Conclusions

DR is one of the serious complications of diabetes and one of the four major blindness diseases in Europe and the United States. Although anti-VEGF therapy is widely used in DR and some retinal diseases, retinal laser photocoagulation remains the main treatment option for these diseases (37). Traditional PRP has some side effects due to the damage to eye tissue caused by laser light. With the advancement of technology, TRP has been continuously applied in clinical practice, and its potential benefits have opened up broad prospects for the treatment of DR. The combination therapy of TRP and anti-VEGF is expected to become a new option for patients with DR and some retinal diseases (such as RVO, radiation retinopathy, etc.). It is believed that with the advancement of science and technology, new treatment methods and approaches will surely bring new hope to DR patients.

## Author contributions

AD and ZL contributed to conception and design of the study. ZL wrote the first draft of the manuscript. NH, LG and XZ wrote sections of the manuscript. All authors contributed to the article and approved the submitted version.

## References

[1] The Early Treatment Diabetic Retinopathy Study Research Group. Techniques for scatter and local photocoagulation treatment of diabetic retinopathy: Early treatment diabetic retinopathy study report no. 3. Int Ophthalmol Clin (1987) 27:254–64. doi: 10.1097/00004397-198702740-00005 3692707

[2] Meyer-Schwickerath GR. The history of photocoagulation. Aust N Z J Ophthalmol (1989) 17:427–34. doi: 10.1111/j.1442-9071.1989.tb00566.x 2696498

[3] Muqit MM Marcellino GR Henson DB Young LB Patton N Charles SJ. Optos-guided pattern scan laser (Pascal)-targeted retinal photocoagulation in proliferative diabetic retinopathy. Acta Ophthalmol (2013) 91:251–8. doi: 10.1111/j.1755-3768.2011.02307.x 22176513

[4] Fong DS Girach A Boney A. Visual side effects of successful scatter laser photocoagulation surgery for proliferative diabetic retinopathy: A literature review. Retina (2007) 27:816–24. doi: 10.1097/IAE.0b013e318042d32c 17891003

[5] Pahor D. Visual field loss after argon laser panretinal photocoagulation in diabetic retinopathy: Full- versus mild-scatter coagulation. Int Ophthalmol (1998) 22:313–9. doi: 10.1023/A:1006367029134 10826550

[6] Henricsson M Heijl A. The effect of panretinal laser photocoagulation on visual acuity, visual fields and on subjective visual impairment in preproliferative and early proliferative diabetic retinopathy. Acta Ophthalmol (Copenh) (1994) 72:570–5. doi: 10.1111/j.1755-3768.1994.tb07181.x 7887154

[7] Reddy SV Husain D. Panretinal photocoagulation: A review of complications. Semin Ophthalmol (2018) 33:83–8. doi: 10.1080/08820538.2017.1353820 29172937

[8] Reddy S Hu A Schwartz SD. Ultra wide field fluorescein angiography guided targeted retinal photocoagulation (TRP). Semin Ophthalmol (2009) 24:9–14. doi: 10.1080/08820530802519899 19241285

[9] Kernt M Cheuteu RE Cserhati S Seidensticker F Liegl RG Lang J. Pain and accuracy of focal laser treatment for diabetic macular edema using a retinal navigated laser (Navilas). Clin Ophthalmol (2012) 6:289–96. doi: 10.2147/OPTH.S27859 PMC3292412 22393280

[10] Muqit MM Young LB McKenzie R John B Marcellino GR Henson DB. Pilot randomised clinical trial of pascal TargETEd retinal versus variable fluence PANretinal 20 ms laser in diabetic retinopathy: PETER PAN study. Br J Ophthalmol (2013) 97:220–7. doi: 10.1136/bjophthalmol-2012-302189 23178855

[11] Takamura Y Tomomatsu T Matsumura T Arimura S Gozawa M Takihara Y. The effect of photocoagulation in ischemic areas to prevent recurrence of diabetic macular edema after intravitreal bevacizumab injection. Invest Ophthalmol Vis Sci (2014) 55:4741–6. doi: 10.1167/iovs.14-14682 25028357

[12] Nikkhah H Ghazi H Razzaghi MR Karimi S Ramezani A Soheilian M. Extended targeted retinal photocoagulation versus conventional pan-retinal photocoagulation for proliferative diabetic retinopathy in a randomized clinical trial. Int Ophthalmol (2018) 38:313–21. doi: 10.1007/s10792-017-0469-7 28168567

[13] Brown DM Ou WC Wong TP Kim RY Croft DE Wykoff CC. Targeted retinal photocoagulation for diabetic macular edema with peripheral retinal nonperfusion: Three-year randomized DAVE trial. Ophthalmology (2018) 125:683–90. doi: 10.1016/j.ophtha.2017.11.026 29336896

[14] Toscano L Messias A Messias K de Cenco Lopes R Ribeiro JAS Scott IU. Proliferative diabetic retinopathy treated with intravitreal ranibizumab and photocoagulation directed at ischemic retinal areas-a randomized study. Doc Ophthalmol (2021) 143:313–22. doi: 10.1007/s10633-021-09848-6 34347216

[15] Oishi A Hidaka J Yoshimura N. Quantification of the image obtained with a wide-field scanning ophthalmoscope. Invest Ophthalmol Vis Sci (2014) 55:2424–31. doi: 10.1167/iovs.13-13738 24667862

[16] Rabiolo A Parravano M Querques L Cicinelli MV Carnevali A Sacconi R. Ultra-wide-field fluorescein angiography in diabetic retinopathy: a narrative review. Clin Ophthalmol (2017) 11:803–7. doi: 10.2147/OPTH.S133637 PMC5415004 28490862

[17] Wessel MM Aaker GD Parlitsis G Cho M D’Amico DJ Kiss S. Ultra-wide-field angiography improves the detection and classification of diabetic retinopathy. Retina (2012) 32:785–91. doi: 10.1097/IAE.0b013e3182278b64 22080911

[18] Cui Y Zhu Y Wang JC Lu Y Zeng R Katz R. Comparison of widefield swept-source optical coherence tomography angiography with ultra-widefield colour fundus photography and fluorescein angiography for detection of lesions in diabetic retinopathy. Br J Ophthalmol (2021) 105:577–81. doi: 10.1136/bjophthalmol-2020-316245 PMC9092310 32591347

[19] Russell JF Flynn HW Jr. Sridhar J Townsend JH Shi Y Fan KC. Distribution of diabetic neovascularization on ultra-widefield fluorescein angiography and on simulated widefield OCT angiography. Am J Ophthalmol (2019) 207:110–20. doi: 10.1016/j.ajo.2019.05.031 31194952

[20] Couturier A Rey PA Erginay A Lavia C Bonnin S Dupas B. Widefield OCT-angiography and fluorescein angiography assessments of nonperfusion in diabetic retinopathy and edema treated with anti-vascular endothelial growth factor. Ophthalmology (2019) 126:1685–94. doi: 10.1016/j.ophtha.2019.06.022 31383483

[21] Russell JF Al-Khersan H Shi Y Scott NL Hinkle JW Fan KC. Retinal nonperfusion in proliferative diabetic retinopathy before and after panretinal photocoagulation assessed by widefield OCT angiography. Am J Ophthalmol (2020) 213:177–85. doi: 10.1016/j.ajo.2020.01.024 PMC7962743 32006481

[22] Ghasemi Falavarjani K Tsui I Sadda SR. Ultra-wide-field imaging in diabetic retinopathy. Vision Res (2017) 139:187–90. doi: 10.1016/j.visres.2017.02.009 28688908

[23] Friberg TR Gupta A Yu J Huang L Suner I Puliafito CA. Ultrawide angle fluorescein angiographic imaging: a comparison to conventional digital acquisition systems. Ophthalmic Surg Lasers Imaging (2008) 39:304–11. doi: 10.3928/15428877-20080701-06 18717436

[24] Shimura M Yasuda K Nakazawa T Abe T Shiono T Iida T. Panretinal photocoagulation induces pro-inflammatory cytokines and macular thickening in high-risk proliferative diabetic retinopathy. Graefes Arch Clin Exp Ophthalmol (2009) 247:1617–24. doi: 10.1007/s00417-009-1147-x 19639332

[25] Soman M Ganekal S Nair U Nair K. Effect of panretinal photocoagulation on macular morphology and thickness in eyes with proliferative diabetic retinopathy without clinically significant macular edema. Clin Ophthalmol (2012) 6:2013–7. doi: 10.2147/OPTH.S37340 PMC3526904 23271879

[26] Diabetic Retinopathy Clinical Research N Brucker AJ Qin H Antoszyk AN Beck RW Bressler NM. 3rd, observational study of the development of diabetic macular edema following panretinal (scatter) photocoagulation given in 1 or 4 sittings. Arch Ophthalmol (2009) 127:132–40. doi: 10.1001/archophthalmol.2008.565 PMC2754061 19204228

[27] Kozak I Luttrull JK. Modern retinal laser therapy. Saudi J Ophthalmol (2015) 29:137–46. doi: 10.1016/j.sjopt.2014.09.001 PMC4398802 25892934

[28] Goel S Kumar A Ravani RD Chandra P Chandra M Kumar V. Comparison of ranibizumab alone versus ranibizumab with targeted retinal laser for branch retinal vein occlusion with macular edema. Indian J Ophthalmol (2019) 67:1105–8. doi: 10.4103/ijo.IJO_1364_18 PMC6611316 31238421

[29] Kwon S Wykoff CC Brown DM van Hemert J Fan W Sadda SR. Changes in retinal ischaemic index correlate with recalcitrant macular oedema in retinal vein occlusion: WAVE study. Br J Ophthalmol (2018) 102:1066–71. doi: 10.1136/bjophthalmol-2017-311475 29699979

[30] Tomomatsu Y Tomomatsu T Takamura Y Gozawa M Arimura S Takihara Y. Comparative study of combined bevacizumab/targeted photocoagulation vs bevacizumab alone for macular oedema in ischaemic branch retinal vein occlusions. Acta Ophthalmol (2016) 94:e225–30. doi: 10.1111/aos.12721 25989706

[31] Yu HJ Fuller D Anand R Fuller T Munoz J Moore C. Two-year results for ranibizumab for radiation retinopathy (RRR): A randomized, prospective trial. Graefes Arch Clin Exp Ophthalmol (2022) 260:47–54. doi: 10.1007/s00417-021-05281-2 34463842

[32] Singer MA Tan CS Surapaneni KR Sadda SR. Targeted photocoagulation of peripheral ischemia to treat rebound edema. Clin Ophthalmol (2015) 9:337–41. doi: 10.2147/OPTH.S75842 PMC4335623 25709396

[33] Gossage DL Cieslarova B Ap S Zheng H Xin Y Lal P. Phase 1b study of the safety, pharmacokinetics, and disease-related outcomes of the matrix metalloproteinase-9 inhibitor andecaliximab in patients with rheumatoid arthritis. Clin Ther (2018) 40:156–165 e5. doi: 10.1016/j.clinthera.2017.11.011 29287749

[34] Finger PT Chin KJ Duvall G. Palladium-103 for choroidal melanoma study, palladium-103 ophthalmic plaque radiation therapy for choroidal melanoma: 400 treated patients. Ophthalmology (2009) 116:790–6, 796.e1. doi: 10.1016/j.ophtha.2008.12.027 19243829

[35] Krema H Somani S Sahgal A Xu W Heydarian M Payne D. Stereotactic radiotherapy for treatment of juxtapapillary choroidal melanoma: 3-year follow-up. Br J Ophthalmol (2009) 93:1172–6. doi: 10.1136/bjo.2008.153429 19414441

[36] Haas A Pinter O Papaefthymiou G Weger M Berghold A Schrottner O. Incidence of radiation retinopathy after high-dosage single-fraction gamma knife radiosurgery for choroidal melanoma. Ophthalmology (2002) 109:909–13. doi: 10.1016/S0161-6420(02)01011-4 11986096

[37] Everett LA Paulus YM. Laser therapy in the treatment of diabetic retinopathy and diabetic macular edema. Curr Diabetes Rep (2021) 21:35. doi: 10.1007/s11892-021-01403-6 PMC8420141 34487257

